# Supplementary figures and images for: Repetitive transcranial magnetic stimulation (rTMS) triggers dose-dependent homeostatic rewiring in recurrent neuronal networks
Source: PLoS Comput Biol. 2023 Nov 13;19(11):e1011027. doi: 10.1371/journal.pcbi.1011027 (PMC10681319; doi:10.1371/journal.pcbi.1011027)

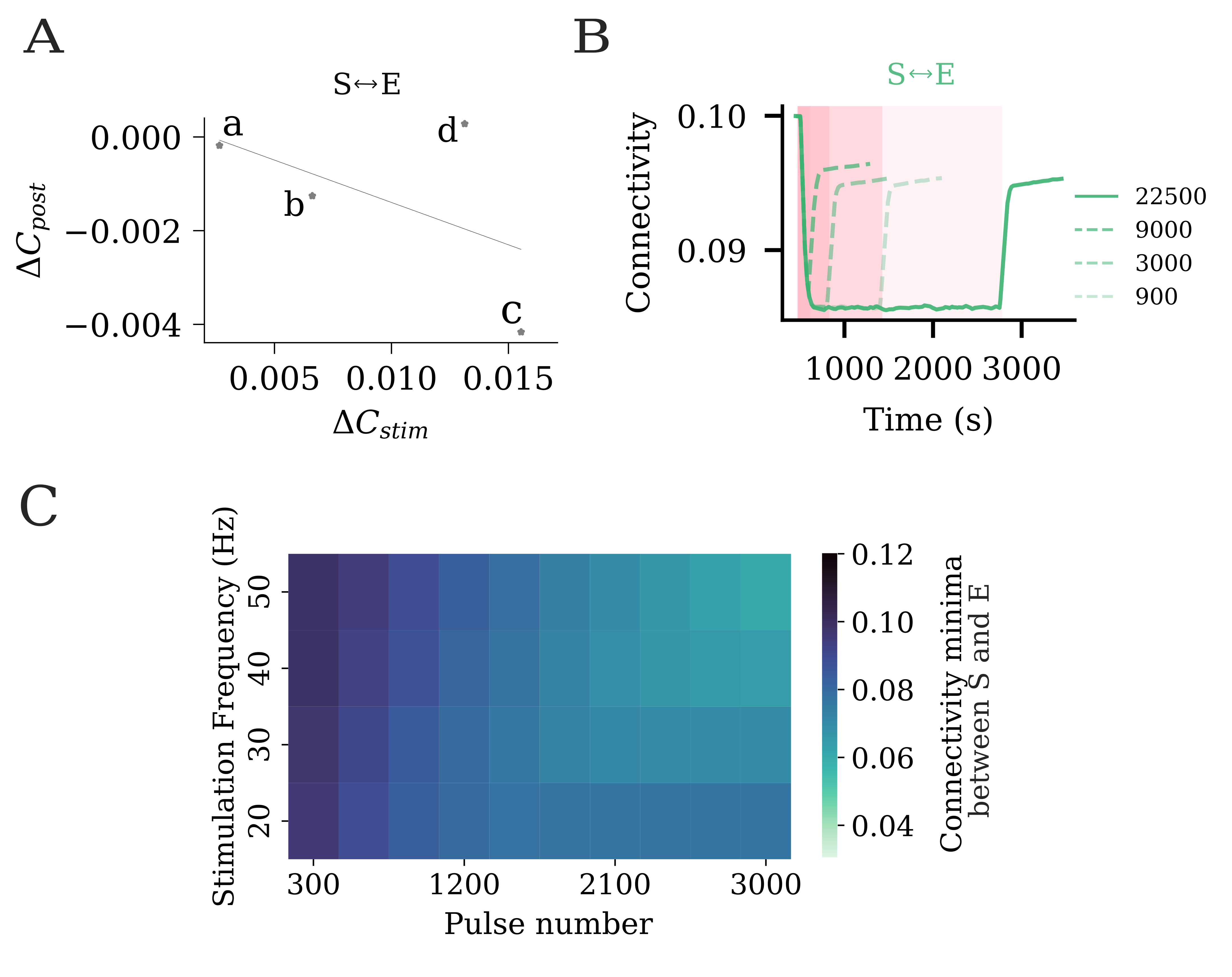

Supplement: S1 Fig — (A) Interrelation between the S − E connectivity drop during stimulation (ΔCstim) and S − E connectivity increase post stimulation (ΔCpost). (B) S − E connectivity changes from different pulse numbers of 10 Hz stimulation at peak stimulation intensity (c, as defined in Fig 2C). (C) Saturation points of S − E connectivity, expressed in the form of total pulse numbers required to reach saturation, are summarized for a range of frequencies. (TIF) [file pcbi.1011027.s001.tif]

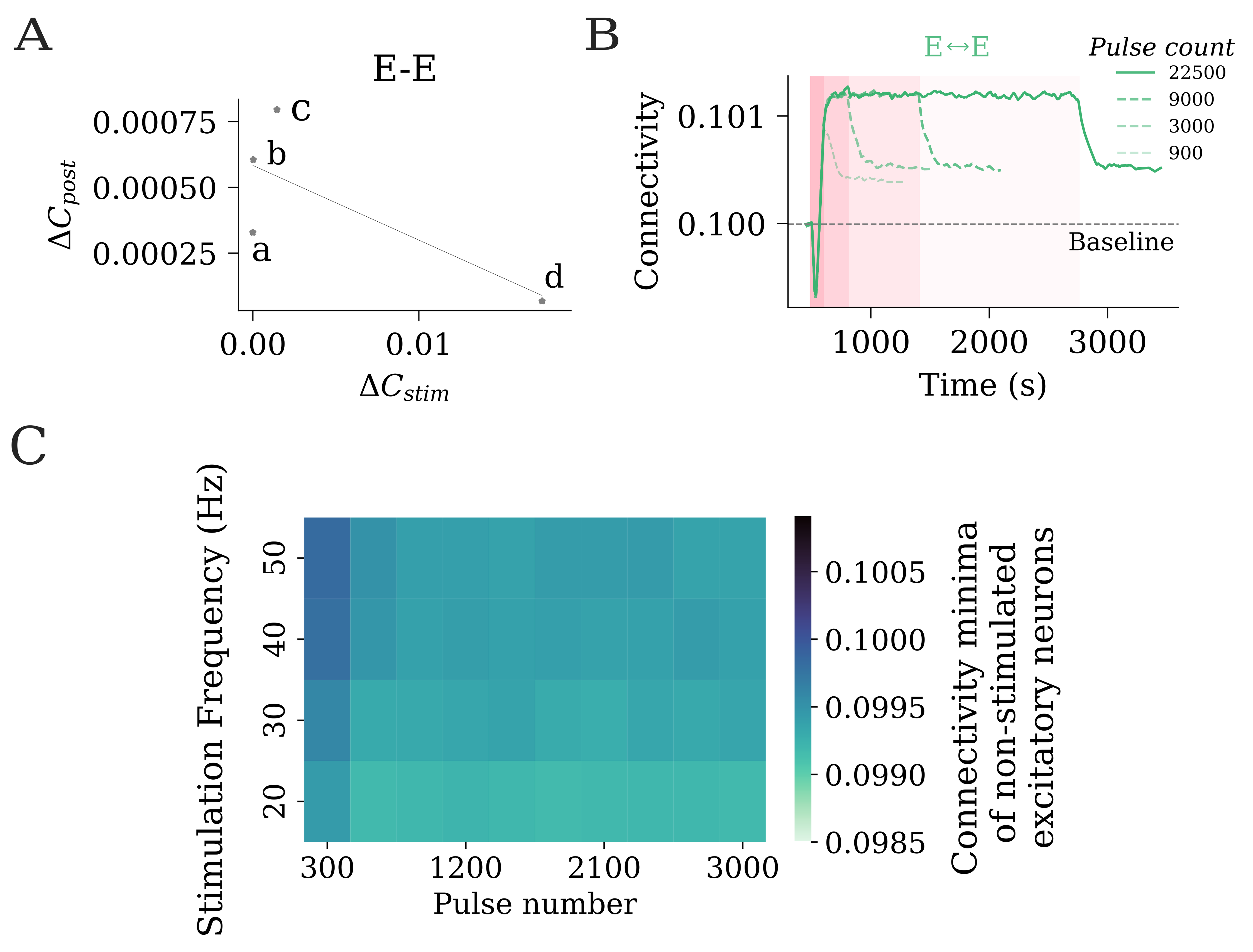

Supplement: S2 Fig — (A) Interrelation between the E − E connectivity drop during stimulation (ΔCstim) and E − E connectivity increase post stimulation (ΔCpost). (B) E − E connectivity changes from different pulse numbers of 10 Hz stimulation at peak stimulation intensity (c, as defined in Fig 2C). (C) Saturation points of E − E connectivity, expressed in the form of total pulse numbers required to reach saturation, are summarized for a range of frequencies. (TIF) [file pcbi.1011027.s002.tif]
