# Supplementary material for: Repetitive transcranial magnetic stimulation (rTMS) triggers dose-dependent homeostatic rewiring in recurrent neuronal networks
Source: PLoS Comput Biol. 2023 Nov 13;19(11):e1011027. doi: 10.1371/journal.pcbi.1011027 (PMC10681319; doi:10.1371/journal.pcbi.1011027)
Supplement: Attachment — Submitted filename: Response to Reviewers_Anil et al..pdf [file pcbi.1011027.s003.pdf]

Point-by-point response:

Reviewer #1:

In this manuscript the authors study effects of repetitive transcranial magnetic stimulation (rTMS) on recurrent networks with homeostatic plasticity. The goal in itself is interesting and worthwhile. The major problem however I have with the manuscript is that the authors use leaky integrate and fire model without any spatial dimension. Is this model at all capturing effects of magnetic stimulation, what about dendritic effects? I understand that things get quickly complicated but at least limitations of this study should be discussed.

Response:

Thank you for pointing at this. We acknowledge limitations of the Leaky Integrate-and-Fire (LIF) model in the revised version of our manuscript:

Lines 570-588 now read: "*In this context, it is crucial to acknowledge that the employed point neuron model falls short in capturing the intricate biophysical characteristics, neuronal diversity, and complex cyto-/fiberarchitecture observed in the brain. Additionally, it fails to accurately represent the various subtypes of neurons, including specific subtypes of principal neurons and interneurons, and their corresponding connectivity patterns. Moreover, the model did not attempt to capture the structural complexities of dendritic and axonal morphologies, leading to limitations in describing cell-type and input-specific effects. As a consequence, the model necessitated relatively high stimulation intensities to elicit action potentials, making it challenging to directly translate these absolute values into realistic effects of single-pulse TMS on individual neurons and small networks. To overcome these limitations and achieve accurate predictions, it is possible to employ multi-scale modeling approaches that incorporate biophysically realistic neurons and account for physiological network activity (Aberra et al., 2018; Shirinpour et al., 2021). Nevertheless, it is important to note that we provided ab initio thinking on how external stimulation should interfere with network dynamics and structural plasticity without being masked by the heterogeneous neural morphology, which could serve as the building blocks in the future to systematically understand the impact of neural morphology in TMS effects.*"

Further, Lines 38-60 now read: "We employed an inhibition-dominated, sparsely connected recurrent neuronal network model using leaky integrate-and-fire point neurons to capture network dynamics influenced by repetitive transcranial magnetic stimulation (rTMS). The model integrates three critical components of cortical networks: asynchronous irregular neuronal activity, inhibition domination, and sparse connectivity. Asynchronous irregular neuronal activity, a distinctive feature of healthy cortical networks, is essential for realistic representation of cortical network dynamics under rTMS. This behavior contributes to neuronal intrinsic properties like stochastic firing, diverse input integration, absence of intrinsic oscillations, and sensitivity to input. Inhibition domination, which underscores the significant role of inhibitory connections in network activity and balance, was included in the model to accurately capture the inhibitory effects on the network dynamics under the influence of rTMS. In an inhibition-dominated network, inhibitory signals help to prevent the network from becoming overly synchronized, which can promote asynchronous firing. Sparse connectivity, a fundamental property of cortical networks where only a small fraction of neurons are interconnected, was incorporated to reflect a realistic cortical network architecture. We opted for a simplistic leaky integrate-and-fire neuronal model, which provided a good balance in terms of computational feasibility while still capturing essential neuronal dynamics relevant to our study. LIF neurons effectively encapsulate asynchronous irregular firing. This ability to replicate irregular and independent firing patterns is particularly crucial to our study, as it involves homeostatic plasticity mechanisms which are likely to induce changes in firing patterns."

Could these results be compared to some experimental ones, like the one referenced in 12? or dissociated cell cultures?

Response:

Our results are most relevant to brain regions that share the characteristics of sparse connectivity, inhibition dominated behavior and asynchronous irregular firing among neurons. These are commonly found across the neocortex in awake animals. Several studies have shown the effect of rTMS in in vivo animal studies. Ma et al (2014) were able to demonstrate that low frequency rTMS plays an important role in the regulation of cognitive behavior accompanied by changes in structural synaptic plasticity. Later, Ma et al (2019) have shown that high

frequency rTMS can alleviate cognitive impairment and modulate hippocampal synaptic structural plasticity in aging mice. Tang et al (2021) also showed that rTMS can drive structural synaptic plasticity in the motor cortex of young and aging mice. However, it remains unclear how specific stimulation parameters may affect structural plasticity of stimulated and non-stimulated brain regions.

We extended our discussion to accommodate additional experimental results related to this study:

Lines 490-511 read: “*In our previous investigation, utilizing live cell microscopy to examine the impact of repetitive transcranial magnetic stimulation (rTMS) on dendritic spines of cultured hippocampal CA1 neurons, we did not observe any significant changes in synapse numbers, including alterations in spine density, following 10 Hz stimulation. This finding aligns with a recent in vivo two-photon imaging study that demonstrated subtle structural changes in dendritic spines in response to repeated sessions of low-intensity rTMS (14). Notably, studies conducted by Ma et al. (2014, 2019) have also shown effects of rTMS on structural plasticity in mice/rats. Homeostatic structural plasticity could potentially involve the (un)silencing of synapses as a biological mechanism. Synapses present on small dendritic spines or filopodia, primarily containing NMDA receptors, are often considered "silent" due to the blockage of NMDA receptors by magnesium ions at the resting membrane potential. However, these synapses can be activated upon the accumulation of depolarizing AMPA receptors. Interestingly, our previous research unveiled that 10 Hz repetitive magnetic stimulation promotes the accumulation of AMPA receptors at existing small spine synapses, triggering the growth of these dendritic spines, which are presumably silent (Vlachos et al., 2012; Lenz et al., 2015). Consequently, rTMS may facilitate homeostatic structural plasticity by enabling neurons to establish or eliminate functional synaptic connections through the regulation of AMPA receptor accumulation at preexisting synapses, without necessitating the recruitment of the complete molecular machinery for the formation or removal of new spines or synapses (c.f., Vardalaki et al., 2022).*”

Reviewer #2:

In their manuscript, “Repetitive transcranial magnetic stimulation (rTMS) triggers dose-dependent homeostatic rewiring in recurrent neuronal networks,” the authors study the effect of stimulation on neuronal networks with and without homeostatic plasticity computationally. The authors find that stimulation of a neuronal subpopulation shapes the firing rate of different neuronal populations in a nonlinear manner. The maximum firing rate is reached at intermediate stimulation amplitudes as stronger stimulation leads to the recruitment of inhibitory neurons, which then reduces the overall firing rate. Stimulation-induced firing rate changes trigger homeostatic plasticity and cause a rewiring of the network. It may also lead to long-lasting changes in the network structure, as quantified by a change of the connection probability.

The paper is very interesting and contains several new results. Since the work is motivated by TMS, it is certainly of interest to the readership of PLoS CB. Furthermore, the paper is well-written. However, there are some major points that the authors need to address before I can recommend publication in PLoS CB. Below I give a detailed description of these major points and some minor changes.

Response:

Thank you for recognizing the value of our work and highlighting its novelty and relevance to the readership of PLoS Computational Biology. We appreciate your commendation.

Major points

1. *The computational network model is not well motivated. Since the authors mean to study TMS, it would be good to state in detail which cortical region is considered and to which extent the network parameters are motivated by experimental data. Of particular importance would be to see whether the firing rates of excitatory and inhibitory neurons and the time scale on which the homeostatic plasticity remodels the network are chosen according to experimental data. I think the time scale of remodeling seems relatively fast (Table 3). What are the time scales on*

which synaptic remodeling occurs in the considered brain region? Furthermore, are the suggested stimulation amplitudes realistic? It seems that very strong stimulation would be required to achieve the suggested changes of the membrane potential.

Response:

We thank the reviewer for these thoughtful comments, which have helped us to improve the clarify and quality of our work.

The Materials and Methods section was extended to provide more information and better motivate the use of a leaky integrate-and-fire point neuron network:

Lines 110-121 now read: "*We implemented a sparsely connected recurrent neuronal network comprising leaky integrate-and-fire point neurons, where inhibition dominated the dynamics (consisting of 10,000 excitatory and 2,500 inhibitory neurons, inhibitory synapses were stronger than excitatory ones). We did not mean to model a specific cortical region, rendering our findings applicable to any neocortical region. Asynchronous irregular neuronal activity, known for its role for recurrent network activity (Brunel, 2000, Tsodyks & Sejnowski, 1995, van Vreeswijk & Sompolinsky, 1996), was faithfully reproduced in our cortical network model, consistent with previous studies (Gallinaro et al, 2018, Lu et al, 2019). While more biophysically detailed models could have been employed, we opted for the leaky integrate-and-fire model to balance between computational feasibility and capturing essential neuronal dynamics relevant to our study. This model allowed us to systematically investigate the effects of rTMS on network dynamics and connectivity with the help of large-scale numerical simulations.*"

Further, lines 66-84 now read; "This rule has been previously demonstrated to have emergent associative properties (Gallinaro & Rotter, 2018). This study showed that homeostatic structural plasticity rule led to the formation of a cell assembly among neurons that receive external stimulation, in absence of explicit correlation-based synaptic plasticity rules. They also showed the emergence of feature-specific connectivity as a result of sensory experience, similar to observations in the V1 region of mice. Additionally, network reorganisation caused by transcranial Direct Current Stimulation (tDCS), another non-invasive brain stimulation technique, has been demonstrated using homeostatic structural plasticity (Lu et al, 2019). This evidence supports the suitability of the

homeostatic structural plasticity rule for capturing the changes in network connectivity induced by rTMS in our model. Moreover, the network characteristics we chose are commonly found across the neocortex in awake animals. Several studies have demonstrated the effect of rTMS in in vivo animal studies. Ma et al (2014) were able to demonstrate that low-frequency rTMS plays an important role in the regulation of cognition-driven behavior by altering the synaptic structure of networks. Later, Ma et al (2019) have shown that high-frequency rTMS can alleviate cognitive impairment and modulate hippocampal synaptic structural plasticity in aging mice. Tang et al (2021) showed that rTMS can drive structural synaptic plasticity in the motor cortex of young and aging mice."

The reviewer has aptly highlighted an essential aspect concerning the time scale of homeostatic structural plasticity. We acknowledge that the precise time scales associated with this process remain uncertain at present. Nonetheless, we introduce the concept of synaptic "un-silencing", which potentially operates on a significantly faster time scale compared to the extensive remodeling of structural connectivity involving synapse formation and elimination.

Lines 511-514 now read: "*While experimental evidence exists supporting the presence of homeostatic structural plasticity (Turrigiano, 2011, Wefelmeyer et al, 2016, Yin et al, 2015), additional research is required to unravel its implementation time scale, biological significance, and the underlying molecular mechanisms.*

And lines 498-511: "*Homeostatic structural plasticity could potentially involve the (un)silencing of synapses as a biological mechanism. Synapses present on small dendritic spines or filopodia, primarily containing NMDA receptors, are often considered "silent" due to the blockage of NMDA receptors by magnesium ions at the resting membrane potential. However, these synapses can be activated upon the accumulation of depolarizing AMPA receptors. Interestingly, our previous research unveiled that 10 Hz repetitive magnetic stimulation promotes the accumulation of AMPA receptors at existing small spine synapses, triggering the growth of these dendritic spines, which are presumably silent (Vlachos et al., 2012; Lenz et al., 2015). Consequently, rTMS may facilitate homeostatic structural plasticity by enabling neurons to establish or eliminate functional synaptic connections through the regulation of AMPA receptor accumulation at preexisting synapses, without necessitating the recruitment of*

the complete molecular machinery for the formation or removal of new spines or synapses (c.f., Vardalaki et al., 2022).”

In our study, we observed that the very brief duration of TMS pulses (0.5 ms) necessitated high amplitudes of the equivalent current to induce suprathreshold membrane potential deviations in our model. However, it is important to note that these specific stimulation amplitude values are not directly translatable. We have made this explicit clarification in the revised version of our manuscript. Thank you for bringing attention to this point.

Lines 575-583 now read: “…Moreover, the model did not attempt to capture the structural complexities of dendritic and axonal morphologies, leading to limitations in describing cell-type and input-specific effects. As a consequence, the model necessitated relatively high stimulation intensities to elicit action potentials, making it challenging to directly translate these absolute values into realistic effects of single-pulse TMS on individual neurons and small networks. To overcome these limitations and achieve accurate predictions, it is possible to employ multi-scale modeling approaches that incorporate biophysically realistic neurons and account for physiological network activity (Aberra et al., 2018; Shirinpour et al., 2021).”

2. *The authors mention that they study an inhibition-dominated network; however, balanced networks are usually studied in the field. Maybe I am missing something here, but why is the network inhibition-dominated? In Figure 5, it seems that the firing rate before stimulation is constant, so isn’t the network in a balanced state before stimulation, where excitation and inhibition balance each other? Please explain the term inhibition-dominated in more detail and discuss how this affects the results.*

Response:

The network must have a surplus of anatomical recurrent inhibition to exhibit a “balanced state”. The latter is a concept from dynamical systems theory, well explained and studied in a paper by Nicolas Brunel (2000). In a previous study using structural plasticity by Gallinaro and Rotter (2018), a plastic inhibition-

dominated network was developed to exhibit asynchronous-irregular network activity. This model serves as a tool for investigating network dynamics and its contribution to information processing in the brain. In our present study, we adopted a similar framework by constructing an inhibition-dominated network under comparable conditions.

Lines 110-116 now read: "*We implemented a sparsely connected recurrent neuronal network comprising leaky integrate-and-fire point neurons, where inhibition dominated the dynamics (consisting of 10,000 excitatory and 2,500 inhibitory neurons, inhibitory synapses were stronger than excitatory ones). We did not mean to model a specific cortical region, rendering our findings applicable to any neocortical region. Asynchronous irregular neuronal activity, known for its role for recurrent network activity (Brunel, 2000, Tsodyks & Sejnowski, 1995, van Vreeswijk & Sompolinsky, 1996), was faithfully reproduced in our cortical network model, consistent with previous studies (Gallinaro et al, 2018, Lu et al, 2019).*"

3. *Although the authors model rTMS, the stimuli are modeled as rectangular current pulses. There are numerous papers studying the effect of electrical stimulation in neuronal networks with STDP, some of which find similar effects of stimulation, i.e., synaptic reshaping of the synapses between stimulated neurons, e.g., Lubenov et al. Neuron 58, 118 (2008) and Kromer and Tass PLoS Comput. Biol. 18, e1010568 (2022), synapses between stimulated and not stimulated neurons Morrison et al. Neural Comput.19, 1437 (2007). The difference seems to be that for STDP the synaptic reshaping depends on the timing between spikes and the transmission delays, whereas the presented results suggest that it mainly depends on the differences in firing rates in networks with homeostatic plasticity. Note that STDP also leads to firing rate-dependent changes of the mean synaptic weights (see Burkitt et al. Neural Comput. 16, 885 (2004)). Are there effects that can be observed in networks with homeostatic plasticity that cannot be observed in networks with STDP? Please compare the observed effects to the results of studies on similar stimulation in networks with STDP. After all, stimulation would likely trigger multiple types of plasticity. In particular, in Kromer and Tass PLoS Comput. Biol. 18, e1010568 (2022), the impact of the stimulation parameters, e.g., the pulse number, the stimulation frequency, and the*

stimulation pattern (e.g., bursting, periodic) was analyzed in detail in networks of LIF neurons with STDP. Are the presented results somewhat comparable to their results? Please discuss

Response:

Homeostatic structural plasticity is robust by design, as it is based on negative feedback. Only very strong and focused stimulation can destabilize network growth (Gallinaro et al., 2022). Recurrent networks with STDP, in contrast, are somewhat sensitive to stimulation, as the plasticity relies on positive feedback between synaptic strength and activity correlations (Morrison et al., 2007). Therefore, it is highly likely that a mechanism similar to homeostatic control is present in plastic brain networks as well. For the present work, we decided to first study the effect of homeostatic control alone, as it already has very interesting properties on the network level (Gallinaro & Rotter, 2018). In future studies, however, we plan to include other forms of plasticity as well.

Kromer and Tass (2022) extensively explored stimulation-induced plasticity from the standpoint of spike-timing dependent plasticity, while our focus has been on homeostatic structural plasticity. We concur that these mechanisms are not mutually exclusive and can concurrently contribute to the effects of rTMS, enriching our collective understanding of this multifaceted technique.

In terms of the methodology used, we recognize that conventional rTMS devices commonly generate sinusoidal waveforms, yet the advancements in stimulator technology now enable the production of more complex waveforms. Our decision to utilize rectangular stimulations was informed by the recognition of the critical role played by the amplitude of the injected current in neuronal firing.

We acknowledge that changes in synaptic weights, both excitatory and inhibitory, can significantly affect the outcome of homeostatic structural plasticity. As part of our ongoing research efforts, we have begun to explore the interaction between homeostatic synaptic scaling and homeostatic structural plasticity (Lu et al., bioRxiv 2023).

We believe our focus on homeostatic plasticity and structural network changes offers a complementary understanding of rTMS-induced plasticity. Through our

computational modelling approach, we have proposed a novel biomechanism for rTMS, showcasing an extensive range of stimulation parameters that highlight the indirect effects of rTMS on inhibitory networks. This perspective introduces the concept of "connectivity saturation".

The primary focus of our study was not to systematically compare the effects and interactions of spike-timing-dependent plasticity (STDP) and homeostatic structural plasticity. Nevertheless, we highly value this comment and have taken it seriously into consideration. As a result, we have made explicit mention of this aspect in our revised manuscript.

Lines 588-599 now read: "*To confirm and expand upon the relevant predictions obtained in our computer simulations, it is essential to develop translational frameworks that integrate computational models with in vitro and in vivo animal studies, as well as experiments conducted on healthy individuals. These combined approaches will enable the validation and extension of our findings on dose-response relationships. In addition, future models should incorporate rTMS-induced synaptic plasticity, encompassing changes in synaptic weights for both excitatory and inhibitory synapses, while considering its interplay with homeostatic structural plasticity (c.f., Kromer and Tass (2022) who conducted a systematic assessment of stimulation parameters in networks of leaky integrate-and-fire neurons with spike-timing-dependent plasticity or Lu et al., (2023) who explored the intricate interplay of homeostatic synaptic scaling and structural plasticity).*"

4. *Panel D of Figure 3 indicates that the studied network is multistable and that TMS can induce transitions between the different stable states. This is reminiscent of works on coordinated reset stimulation, where the goal was to induce a transition from a pathological state to a physiological (Manos et al. Frontiers in Physiology 12, 716556 (2021)). Based on the presented results, would TMS be able to induce such a transition? Please discuss.*

Response:

Thank you for this interesting comment.

What is shown in Figure 3D is the outcome of 4 different stimulation experiments (a–d). More details can be retrieved from Figure 3 in Gallinaro & Rotter (2018). The result of each such stimulation experiment is a substantial rewiring, leading to different activity levels as a result of stimulation. One should not speak of "multistability" here. Each of the networks, in every moment, has a unique balanced state that determines its activity. The above-mentioned 2018 paper also depicts spike trains that underpin this interpretation. Therefore, one should also not speak of induced "transitions between different stable states", and the rewiring is essentially irreversible.

It is worth noting that the DBS field has made significant progress in this regard, with a more advanced understanding on the effects of stimulation on complex network dynamics. In contrast, the design of rTMS protocols still heavily relies on studies conducted in the motor cortex, utilizing indirect measures of long-term potentiation (LTP) or long-term depression (LTD)-like plasticity.

As our study primarily focuses on non-pathological brain states, we have chosen not to delve into a detailed discussion of pathological aspects at this stage. Nonetheless, we recognize the significance of these intriguing issues and consider them as promising avenues for future exploration. In our upcoming endeavors, we intend to collaborate with leading experts in this field, leveraging their expertise and insights to further investigate the implications of our findings in the context of pathological brain states.

We now write (Line 612-621): "*We have confidence that these computational models will play a crucial role in evaluating the effects of rTMS under disease conditions, thereby informing protocol designs in clinical practice (c.f., Manos et al., 2021). Currently, in the field of TMS these designs heavily depend on studies that report rTMS-induced long-term potentiation (LTP) or long-term depression (LTD) like plasticity in the motor cortex and do not effectively consider the intricate dynamics and states of complex neural networks and their relationship with structural plasticity. By incorporating these computational models, we can better understand and optimize the effects of rTMS, paving the way for more effective therapeutic interventions.*"

5. *Equation 3 is unclear to me. In line 109 z_i^k, was introduced as a "discrete number," however, in Equation 3, it seems to be a (positive) real number. How are z_i^k and the numbers of dendritic spines and axonal boutons related? Furthermore, I guess k should be either "a" or "d" (lines 109 and 110) and not "pre" or "post" (Equation 3). Please clarify the notation and the definition of z_i^k.*

Response:

Thank you for pointing this out. The rewiring process is actually a Markovian jump process. We approximate this discrete process as a continuous movement, given by the solution of an ordinary differential equation. Please see Gallinaro et al. (2022) for more details, if you are interested. We use the notation z_i^k to represent synaptic elements, where k can be 'pre', denoting a presynaptic element (axonal boutons), or 'post', denoting a postsynaptic element (dendritic spines). In order to clarify this, Lines 159-161 now read:

"Each neuron i in this model has a number of available axonal boutons (presynaptic elements, z_i^{pre}) and dendritic spines (postsynaptic elements, z_i^{post}), which are paired to form functional synapses."

We also have replaced "discrete" with "available".

6. *In Figure 4, the overall drop in connectivity is studied. I wonder whether this is the best quantity to study. Wouldn't it yield more insight if one would distinguish between the connection probability between the stimulated subpopulation and the other subpopulations? Please elaborate.*

Response:

We appreciate this comment. Although our primary investigation has centered on the connectivity between stimulated neurons, we have included supporting information regarding the connectivity outcomes for the S-E and E-E subpopulations in the revised version of our manuscript. Figures S1 and S2 illustrate that the conclusions drawn for the S-S subpopulation remain consistent for the other subpopulations as well.

Minor

7. *Throughout the paper: Please increase the font size in the figures. In some cases, it is very difficult to read the labels in the figures.*

Response:

Done!

8. *Line 57: "spike trains" -> "spike train"*

Response:

Corrected!

9. *Line 67: Why is the reset potential above the resting potential? This does not seem to be the case in Fig. 1D.*

Response:

The values of reset, resting and threshold values have been corrected in the revised manuscript. We apologize for the error (delta values were shown in the original submission). Accordingly, the resting potential is -60mV, the reset potential is -50mV and threshold membrane potential is -40mV. In our model, when the membrane potential reaches the value of the firing threshold, the neuron emits a spike and its membrane potential is then reset to -50mV, and is kept at that value for the refractory period of 2ms.

10. *Line 80: What is the motivation for the asynchronous-irregular resting state? Is this motivated by a certain cortical region? What other states exist in the network?*

Response:

The neurons in the network maintain an asynchronous irregular firing rate. A disturbance, for example by rTMS, causes the firing rates of stimulated neurons to elevate. By virtue of the recurrent nature of network connections, the firing rate of non-stimulated neurons is also affected.

The changes in firing rate thus caused are restored back to the AI state with the help of homeostatic structural plasticity mechanisms. In the present study, we do not observe other states of neuronal activity, see Gallinaro & Rotter (2018) for more information on this issue.

Our choice of this network features was not restricted to a specific cortical region, rendering our findings applicable to various neocortical regions.

Lines 43-48 now read: "Asynchronous irregular neuronal activity, a distinctive feature of cortical networks, is essential for realistic representation of neuronal activity dynamics under rTMS. This behavior contributes to neuronal intrinsic properties like stochastic firing, diverse input integration, absence of intrinsic oscillations, and sensitivity to input."

11. *Table 2: Please distinguish between static and plastic networks.*

Response:

The caption of Table 2 has been updated to indicate that the table summarizes characteristics that apply to both static and plastic networks: "Parameters of static and plastic network models". Additional characteristics of the plastic network are included in Table 3, captioned: "Parameters of structural plasticity model"

12. *Line 87: "equilibrium status" I guess this should be "equilibrium state".*

Response:

Corrected!

13. *Line 110: "axonal buotons" -> "axonal boutons"*

Response:

Done!

14. *Line 122: "by amount, beta..." -> "by beta..."*

Response:

Thank you!

15. *Line 145: "modeled after output of conventional rTMS devices" Please double-check whether this sentence is correct.*

Response:

Line 196 now reads: "..inspired by the output of conventional rTMS devices"

16. *Lines 150, 151: "cite"-> "site"*

Response:

Done!

17. *Line 160: "The effect of rTMS over networks" I guess this should be "The effect of rTMS on networks" instead.*

Response:

Correct and corrected!

18. *Line 169: "protocol structure" This term is unclear to me. Please explain what this term refers to.*

Response:

Line 196 now reads: "protocol design". The correction has been made to all the instances in the manuscript as well. The phrase "protocol design" refers to the core properties of a stimulation protocol, which include, among others, frequency, total pulse number, intensity and intertrain intervals. These properties strongly influence the outcome of stimulation, in terms of induced changes in brain excitability.

19. *Line 171: FDA-approved for what?*

Response:

The iTBS protocol has been approved by the US FDA for treating pharmacoresistant Major Depressive Disorder (MDD). Lines 208-210 now read: "This protocol has been approved by the US Food and Drug Administration (FDA) for treating pharmacoresistant Major Depressive Disorder (MDD) (Cohen et al, 2022)."

20. *Line 175: "rTMS pulse triggering membrane potential deviation" I guess this should be "rTMS pulse-triggered membrane potential deviation" instead.*

Response:

Line 213 now reads: "rTMS pulse-triggered membrane potential deviation"

21. *Line 184: "nonidentical but equal net Poisson inputs" This term is unclear to me. Are you talking about different realizations of the Poisson inputs here?*

Response:

We used 500 isolated neurons, each receiving independent Poisson inputs of the same rate. The clarification has been added to the manuscript. Line 220-222 now reads: "In order to account for randomness and variability, we traced membrane potentials of 500 isolated neurons, each receiving statistically independent Poisson inputs of the same rate. "

22. *Line 192 and later in the manuscript: "followed by an 8s interval" Interval of what? I guess the authors mean an 8s pause or an 8 second inter-burst interval. Please clarify throughout the manuscript.*

Response:

The term interval refers to a pause in stimulation. Line 230 now reads: "The pulses are delivered in the theta burst format for 2s, followed by an 8s pause. This cycle is repeated 20 times"

23. *Throughout the manuscript: "integrate and fire" -> "integrate-and-fire"*

Response:

Thank you. Done!

24. *The discussion of the Ohms' Law on page 7 is a little confusing. How are U,A, and R related to the integrate-and-fire model in Equation 1?*

Response:

The section that describes Ohm's Law, 'Estimation of membrane potential deviation using Ohm's Law', has been adapted to make it more relatable to Equation 1. Equation 4 has been reformulated to express the terms relevant to the neurons.

25. *Line 205: "formulation" I guess this should be "equation" instead.*

Response:

Corrected!

26. *Line 210: "across defined time-steps" This is unclear to me. Is the firing rate based on the spike count in a 1000s interval?*

Response:

The firing rate is determined based on the average spike count within 1000ms intervals, as a spike count average. Lines 248-251 now read: "*The firing rate is then determined based on the average spike count within 1000ms intervals, as a spike count average. The average firing rate for a population is determined by calculating the arithmetic mean of the individual firing rates of all neurons within that population.* "

27. *Line 211: "from the group"->"in this population"*

Response:

Corrected as correctly requested!

28. *Line 218: "were" -> "was"*

Response:

Thanks!

29. *Line 219: "mean of the whole matrix" I would write "mean number of synapses between two neurons".*

Response:

Thank you for the suggestion. Line 259 now reads: "It is thus calculated as the mean number of synapses between two neurons."

30. *Line 239: “repeated” -> “repetitive”?*

Response:

This suggestion has been implemented.

31. *Line 242: “experimental setting” Please write “simulation” instead. The authors did not perform experiments.*

Response:

Done!

32. *Figure 2: Please clarify whether homeostatic plasticity is ON or OFF in this network. Please also clarify for which population of neurons the firing rate is shown in Figure E.*

Response:

Figure captions and legends have been updated to implement these clarifications. The caption now reads: “Repetitive transcranial magnetic stimulation (rTMS) changes network activity in a static network.” And the legend text corresponding to Fig 2E reads: “Heatmaps summarizing the response of stimulated neurons to rTMS applied to 10% (top) and 30% (bottom) of excitatory neurons.”

33. *Throughout the paper: Please write “different stimulation protocols” instead of “distinct stimulation protocols”*

Response:

Thank you!

34. *Line 345: It is unclear which “two parameters” the authors are talking about here.*

Response:

The two parameters referred to here are:

A. Changes in network connectivity following stimulation
B. Degree of reorganization induced during the stimulation process

Lines 386-391 now read: “This observation had important implications for the stimulation duration, including the number of pulses applied at a given frequency. The finding suggests that once the firing rate of stimulated neurons is restored via network reorganization during stimulation, the application of additional pulses will not have a further effect on the outcome of the intervention, at least not in terms of lasting changes in network connectivity after stimulation.”

35. *Lines 347-350: Please specify which neuronal population is considered here.*

Response:

Here we consider the stimulated population (S). Lines 387-391now read: “The finding suggests that once the firing rate of stimulated neurons is restored via network reorganization during stimulation, the application of additional pulses will not have a further effect on the outcome of the intervention, at least not in terms of lasting changes in network connectivity after stimulation.”

36. *Figure 3B: It is unclear to me whether the firing rate saturates here. Also, please write “Poisson input” instead of “Poissonian input”.*

Response:

Thank you for this careful observation. We extend the graph to 750 s. See revised Figure 3.

37. *Lines 364-365: Please rewrite. What does the first part of the sentence have to do with the second part? It is unclear which “connectivity” is studied here? Is this the connection proability? Please clarify.*

Response:

Lines 405-407 now read: “Considering that the pulse number is a product of stimulation duration and frequency, it is useful to evaluate the impact of stimulation duration on connectivity saturation as well.”

38. *Line 408-410: Optimality with respect to what? Is the goal here just to increase the firing rate of the stimulated neuronal population? Please clarify.*

Response:

Thank you for this comment. We acknowledge that the term 'optimal' here indeed is misleading. Line: 451-453 now reads: "Thus, stimulation protocols may exist, where no additional desired effects will be observed by further increasing the intensity of stimulation or number of TMS pulses."

39. *Line 445: "overview"->"a review article"*

Response:

Done!

40. *Line 463: "need to recruit" -> "need for recruiting"?*

Response:

Thank you very much!

41. *Line 495: Please write "may initiate homeostatic synaptic remodeling".*

Response:

Done!

42. *Line 498: What are the "affected neurons"? Are this the stimulated neurons?*

Response:

Thank you for the comment. For the sake of clarity, Line 556-557 now reads: "The end of stimulation period is followed by further synaptic remodeling causing increase in connectivity among the stimulated neurons."

We are grateful to this reviewer for his/her invaluable contributions and the dedicated time invested in thoroughly reviewing our manuscript. The insightful comments have significantly contributed to enhancing the overall quality of our work.

Reviewer #3:

Based on previous computational modeling studies, the authors introduce a model to describe the effect of rTMS-like stimulation on the reorganization of cortical connectivity. This approach is interesting and modeling studies could indeed help to better understand rTMS. However, in the current state of the manuscript, the link to rTMS remains superficial. Furthermore, although the idea of structural plasticity is very appealing, the authors miss to show how it can be distinguished from the state-of-the-art LTP/LTD-hypothesis. Unfortunately, also some model assumptions stay unclear.

Major Points:

1. *Cortical circuits show many different types of activity-dependent plasticity mechanisms and it is unclear which mechanisms are triggered by rTMS. With this model at hand, I am wondering how the network changes induced by structural plasticity differ from changes induced by LTP/LTD or other mechanisms. Such investigations could also lead to specific hypotheses that could be tested to verify whether structural plasticity is the main process driving TMS-dependent network reorganization. But for this, also the next point has to be clarified.*

Response:

Thank you for your insightful comments and highlighting the important question regarding the specific mechanisms triggered by rTMS in cortical circuits. We agree that understanding how the network changes induced by structural plasticity differ from those induced by long-term potentiation (LTP), long-term depression (LTD), or other mechanisms is an interesting area for investigation.

With our developed computational model, we have a unique opportunity to investigate the effects of homeostatic structural plasticity on network dynamics and connectivity. This exploration is motivated by two key factors. Firstly, the transient nature of LTP- and LTD-like plasticity, which typically lasts for minutes to hours, stands in contrast to the prolonged therapeutic after-effects rTMS that can persist for weeks or even months. Secondly, considerable inter- and intra-individual variability has been reported in the field regarding the induction of LTP

and LTD, suggesting that therapeutic effects may not necessarily depend on the induction of Hebbian plasticity immediately after stimulation.

In our study, we specifically focused on exploring lasting effects on connectivity that can occur without changes in synaptic weights. However, we acknowledge the need for further investigation to fully understand the distinct contributions of structural plasticity in comparison to other forms of plasticity. It is important to recognize that functional and structural changes are likely to interact, although it is likely that these mechanisms may operate independently at different time scales.

To contextualize our findings within the existing literature and establish a connection between LTP induction and homeostatic structural plasticity, we discuss this relationship in lines 498-514 of the revised manuscript.

"*Homeostatic structural plasticity could potentially involve the (un)silencing of synapses as a biological mechanism. Synapses present on small dendritic spines or filopodia, primarily containing NMDA receptors, are often considered "silent" due to the blockage of NMDA receptors by magnesium ions at the resting membrane potential. However, these synapses can be activated upon the accumulation of depolarizing AMPA receptors. Interestingly, our previous research unveiled that 10 Hz repetitive magnetic stimulation promotes the accumulation of AMPA receptors at existing small spine synapses, triggering the growth of these dendritic spines, which are presumably silent (Vlachos et al., 2012; Lent et al., 2015). Consequently, rTMS may facilitate homeostatic structural plasticity by enabling neurons to establish or eliminate functional synaptic connections through the regulation of AMPA receptor accumulation at preexisting synapses, without necessitating the recruitment of the complete molecular machinery for the formation or removal of new spines or synapses (c.f., Vardalaki et al., 2022). While experimental evidence exists supporting the presence of homeostatic structural plasticity (Turrigiano, 2011, Wefelmeyer et al, 2016, Yin et al, 2015), additional research is required to unravel its implementation time scale, biological significance, and the underlying molecular mechanisms.*"

2. *What is the clinical/functional meaning of the observed connectivity remodeling? Is more remodeling better or worse? Or is a specific type of remodeling being needed? At the moment, it remains unclear how to relate the results from the model to results from rTMS-studies.*

Response:

Thank you for raising these important questions regarding the clinical and functional implications of the observed connectivity remodeling and how it relates to the findings from rTMS studies. We acknowledge that understanding the clinical significance of the observed changes in connectivity is crucial for translating our model results into meaningful insights for rTMS applications.

Studies have demonstrated that targeted rTMS interventions can modulate connectivity patterns and alleviate symptoms in patients. For example, in patients with PTSD, rTMS targeting specific brain regions has shown promising results in modifying connectivity and improving clinical outcomes (Petrosino et al., 2021). Similarly, in depression, rTMS-induced connectivity changes have been associated with improved mood and clinical response (Schiena et al., 2021). This now mentioned in the revised version of our manuscript.

Regarding the question of whether more remodeling is better or worse, we should consider the network dynamics output and behavioral relevance. Stronger remodeling, i.e., higher resulting connectivity among the stimulated neurons (clustering), which leads to higher triggered activity as shown by Gallinaro et al. (2023), could be desirable in scenarios where elevated local neural activity is needed. Nevertheless, such effects on network dynamics could also be achieved or mediated by functional plasticity, such as STDP. Therefore, plasticity in health and disease is generally termed as adaptation and maladaptation, depending on its impact on behavioral outcomes. On the other hand, it is worth noticing that network remodeling is an ever-changing process that external stimulation may induce increased connectivity and cell assembly formation, while the cell assembly also decays over time, although at a much lower time scale (Gallinaro et al., 2018, 2022; Lu et al., 2019). Furthermore, our simulation results clearly indicate that a higher stimulation intensity or pulse number will not necessarily yield a better outcome. This finding highlights the need for careful selection of

stimulation parameters, as excessive stimulation may not necessarily lead to improved clinical results.

As for the question of specific types of remodeling that are needed, it is currently challenging to provide a definitive answer. Similar to the current protocol design in clinical practice, which primarily addresses LTP- or LTD-like plasticity, the identification of specific remodeling requirements remains an area of ongoing research. Dose-response curves for rTMS-induced connectivity changes are not yet established (as is the case for LTP/LTD), and this also applies to determining the optimal parameters for stimulation paradigms.

We think that computational models have the potential to bridge these gaps and aid in optimizing stimulation paradigms by providing insight into the complex relations between stimulation parameters, plasticity mechanisms, and expected clinical outcomes.

Lines 588-607 of the revised manuscript now read: "*To confirm and expand upon the relevant predictions obtained in our computer simulations, it is essential to develop translational frameworks that integrate computational models with in vitro and in vivo animal studies, as well as experiments conducted in healthy individuals. These combined approaches will enable the validation and extension of our findings on dose-response relationships. In addition, future models should incorporate rTMS-induced synaptic plasticity, encompassing changes in synaptic weights for both excitatory and inhibitory synapses, while considering its interplay with homeostatic structural plasticity (c.f., Kromer and Tass (2022) who conducted a systematic assessment of stimulation parameters in networks of leaky integrate-and-fire neurons with spike-timing-dependent plasticity or Lu et al., (2023) who explore the intricate interplay of homeostatic synaptic scaling and structural plasticity). We have confidence that these computational models will play a crucial role in evaluating the effects of rTMS under disease conditions, thereby informing protocol designs in clinical practice (c.f., Manos et al., 2021). Currently, in the field of TMS these designs heavily depend on studies that report rTMS-induced long-term potentiation (LTP) or long-term depression (LTD)-like plasticity in the motor cortex and do not effectively consider the intricate dynamics and states of complex neural networks and their relationship with structural plasticity. By incorporating these computational models, we can better*

understand and optimize the effects of rTMS, paving the way for more effective therapeutic interventions."

3. *Eq. 3: The authors are considering the same rule for the growth of axonal and dendritic elements. However, in literature, several models consider that axonal elements behave different to dendritic ones. How critical is this assumption of same rules?*

Response:

Thank you for your insightful comment on Equation 3, which assumes a uniform growth rule for both dendritic and axonal elements. In the large-scale network model, we aimed to simplify while capturing key aspects of plasticity, hence the adoption of a common rule. Although biological evidence suggests potential differences in axonal and dendritic growth behavior, we believe that our findings are fundamentally robust for this class of models. The potential variability in dendritic and axonal growth is anyway dominated by the overall network growth in our analysis, as we focus on the changes in network connectivity following neuronal perturbations. We believe that the observed effects in our model are a result of global dynamics rather than the specific dendritic or axonal behaviours.

However, we recognize the importance of fine-grained biological realism and we look forward to refining our model in future work to include distinct growth rules for dendritic and axonal elements.

We thank you for directing our attention to this matter and for fostering a more nuanced discussion of our model's assumptions and their implications.

4. *Throughout the manuscript the notation is sometimes confusing. In Eq. 1 the impact of rTMS is given by \Delta V_rTMS. In Table 4 is written \Delta V and in the results section is written \Delta V_m. Are all these parameters the same?*

Response:

Thank you for pointing this put. All the said Instances have been replaced by \Delta V_rTMS.

5. *Figure 1B,C: I am confused here. In Eq. 1, the authors introduce that rTMS stimulation induces directly a change in membrane potential. However, here they introduce a current I(t). What is the relation? How is panel C linked to panel B?*

Response:

Δv_m in Fig 1B was measured using a voltmeter in NEST. In Fig 1C, we use two methods to find the value of Δv_m: measure using voltmeter (Numerical simulation) or calculated using Ohm's Law, as explained in Eq. 4 in Section 'Estimation of membrane potential deviation using Ohm's Law'.

Since the neurons received balanced excitatory and inhibitory Poisson input and were isolated, the membrane potential changes caused were essentially due to rTMS. Therefore, in this scenario,

$$\Delta v_m \propto \Delta v_{rTMS}$$

To describe this in a clearer way, the legend of Figure 1 now reads: "(A) Schematic illustration of TMS in humans and neurons. The TMS-induced electric fields cause depolarization of neurons in the target region. We implemented TMS as rectangular pulse current injections with a duration of 0.5 ms (c.f., standard output parameters of conventional TMS devices). (B) Single stimuli produce changes in the membrane potential in a dose-dependent manner. (C) A linear relationship can be seen between applied effective stimulation strength (in nA) and membrane potential deviation caused, as predicted by Ohm's law (See Methods). (D) Suprathreshold stimulation at different frequencies elicits spiking responses from the stimulated neurons."

6. *Why should a rTMS stimulation depolarize only excitatory neurons? I would also expect that some inhibitory neurons are affected. Would this change the results?*

Response:

We acknowledge that our study considers excitatory neurons as a primary target of rTMS and that possible direct effects on interneurons are currently not

considered. While we cannot not exclude direct effects on interneurons, the assumption that principal neurons are a major target is well supported by experimental data. For example, the study from Mathew Larkum's lab (Murphy et al., 2016) demonstrated that TMS directly activates excitatory axons in superficial layers, leading to activation of dendrite-targeting inhibitory neurons, which in turn suppress dendritic Ca2+ activity in pyramidal neurons. This observation aligns very well with our simulations, which show increased inhibition at higher stimulation intensities.

A systematic assessment of the direct effects of single pulse and rTMS on specific interneuron types (e.g., PV or SOM-expressing interneurons) was beyond the scope of the present study and would have required a different modeling approach, i.e., multi-scale modeling using biophysically realistic single-cell compartmental models.

Lines 529-552 of the revised manuscript read: "*Experimental evidence suggests that single-pulse TMS exerts inhibitory effects on neocortical dendrites by directly activating axons in the upper cortical layers, subsequently leading to the activation of dendrite-targeting inhibitory neurons within the neocortex of mice. Our previous experimental research has further demonstrated that 10 Hz rTMS induces remodeling of inhibitory synapses. Specifically, we observed reductions in dendritic inhibition, as well as changes in the strength, sizes, and numbers of inhibitory synapses onto pyramidal neurons following stimulation. These findings underscore the structural modifications occurring in inhibitory networks as a result of rTMS. Notably, rTMS has also been shown to trigger the remodeling of visual cortical maps. However, the direct effects of stimulation on inhibitory neurons and the role of homeostatic structural plasticity in inhibitory synapses remain elusive. To gain a deeper understanding of these dynamics, further investigation is needed to explore the dose-dependent effects on specific inhibitory neuron types and their implications for rTMS-induced structural remodeling of excitatory and inhibitory synapses. These considerations highlight the importance of investigating the direct impact of stimulation on inhibitory neurons and elucidating the interplay between inhibitory synaptic plasticity and homeostatic structural plasticity. Importantly, our findings suggest that stronger stimulation may result in less effective structural remodeling of stimulated networks compared to weaker stimulation that produces equivalent changes in*

firing rates. This highlights the complexity of the relationship between stimulation parameters, network dynamics, and structural plasticity. Further research is warranted to fully unravel these relationships and optimize stimulation protocols for achieving desired structural remodeling outcomes."

Minor Points:

7. *Line 67: The reset potential is noted by V_reset and in table 1 by V_r. Also, why is the reset potential higher than the resting potential? Often set it below the resting potential to emulate the refractory period.*

Response:

The nomenclature has been re-defined:

Reset Potential : V_reset

Resting Potential: V_rest

8. *How does r_ext goes into Eq. 1?*

Response:

The term "r_ext" in our model refers to the rate of external input that is provided to the neurons. Specifically, it represents a Poisson spike train with a fixed frequency (30 kHz), which emulates background input to the neurons. In Equation 1, the effect of "r_ext" manifests as spikes generated within the neurons. However, it's important to note that the spike count (S) is not solely determined by the external input but is also influenced by the recurrent input from other neurons within the network. This interplay between external and recurrent inputs contributes to the overall spike activity and dynamics observed in our model.

9. *Lines 153-168: These paragraphs should be moved to the discussion section.*

Response:

Thank you. Lines 480-483 now read: “Previously, Gallinaro and Rotter (2018) demonstrated emergent associative properties of homeostatic structural plasticity, via activity-driven formation of neuronal ensembles (35). A similar modeling approach has been used to explore effects of transcranial direct current stimulation (Lu et al., 2019)."

10. *Fig. 1D: How can the membrane potential without stimulation be at -60 mV if the resting potential is at 0mV? In addition, after a spike, in these plots the reset is even below -60 mV, although it is 10 mV.*

Response:

The values of reset, resting and threshold values have been corrected in the revised manuscript. We apologize for the error (delta values were shown in the original submission). Accordingly, resting potential is -60mV, reset potential is -50mV and threshold membrane potential is -40mV. In our model, when the membrane potential reaches the value of the firing threshold, the neuron emits a spike and its membrane potential is then reset to -50mV, and is kept at that value for the refractory period of 2ms.

11. *Fig. 2: What is the time axis in panel B? At which point of time after or during stimulation do you evaluate the firing rate to obtain panel C? a, b, c, and d are lines and not points in panel C.*

Response:

Fig 2B has been replaced with a version that includes timestamps.

Fig 2C The traces in this graph represent average firing rate of a neuronal population during an rTMS protocol. These rTMS protocols can be defined by frequency, pulse number and intensity. The rTMS intensity is represented using the membrane potential deviation caused by single rTMS pulses. Accordingly, point **a** indicates the average firing rate of stimulated excitatory subpopulation (S) during rTMS of 10Hz, 900 pulses and intensity a=weak.

For more clarity, the legend of Figure 2 now reads:

"*Repetitive transcranial magnetic stimulation (rTMS) changes network activity in a static network. (A) Illustration of the recurrent neuronal network with sparsely connected excitatory [E] and inhibitory [I] neurons used in this study. A subset of excitatory neurons [S] is stimulated. (B) rTMS influences the firing state of the stimulated neurons [S], causing them to fire in a more synchronous manner. (C) Change in the average firing rate in response to different stimulation intensities and frequencies of 10% of excitatory neurons. Four intensities (a: weak, c: peak, d: strong, and b: strong-equivalent) were selected to represent different stimulation intensities. (D) Firing rate histograms for populations E, I, and S at stimulation intensities a, b, c, and d, respectively. (E) Heatmaps summarizing the response of stimulated neurons to rTMS applied to 10% (top) and 30% (bottom) of excitatory neurons.*"

The code can can be found here: https://github.com/swathianil/homeostatic_structural_plasticity_rTMS
