# Supplementary material for: Repetitive transcranial magnetic stimulation (rTMS) triggers dose-dependent homeostatic rewiring in recurrent neuronal networks
Source: PLoS Comput Biol. 2023 Nov 13;19(11):e1011027. doi: 10.1371/journal.pcbi.1011027 (PMC10681319; doi:10.1371/journal.pcbi.1011027)
Supplement: Attachment — Submitted filename: Revision2_ResponseLetter.docx [file pcbi.1011027.s004.docx]

*Point-by-point response:*

**Reviewer #1**

The authors disused in depth the limitations of the model and compared their results more closely to experimental ones. I believe the manuscript is improved and can be now published.

Thank you for your positive feedback and for recognizing the efforts we have put into addressing the limitations of our model and aligning our results more closely with experimental data. We are pleased to hear that you find the revised manuscript improved and suitable for publication. We appreciate your time and constructive insights throughout the review process, and we are eager to see our work reach a wider audience.

**Reviewer #2**

The authors addressed most of my comments thoroughly. I only have a few further minor points, which I list below. Once these points have been addressed, the manuscript is suitable for publication. I do not need to see the manuscript again before publication.

Thank you for acknowledging the efforts we have made to address your comments comprehensively. We appreciate your detailed feedback and are committed to making the necessary adjustments to address the minor points you have listed below. We are encouraged by your support for the publication of our manuscript once these points are addressed. We value your time and expertise throughout this review process and are grateful for your willingness to approve the manuscript for publication without further review. We look forward to making the final adjustments and moving towards publication. Thank you once again for your constructive feedback.

Lines 45-46:
"This behavior contributes to neuronal intrinsic properties like stochastic firing, diverse input integration, absence of intrinsic oscillations, and sensitivity to input." It needs to be clarified what behavior the authors refer to. Reading this sentence, I would understand that "behavior" refers to the "asynchronous irregular neuronal activity," which would be a network property, and I would not consider a network property to contribute to the intrinsic properties of the neurons. My suggestion would be to write something along the lines of "The dynamics of individual neurons is characterized by stochastic firing, diverse input integration, absence of intrinsic oscillations, and sensitivity to input."

Thank you for the suggestion and the sentence has been rephrased accordingly. Lines 45-47 now read “The dynamics of individual neurons is characterized by stochastic firing, diverse input integration, absence of intrinsic oscillations, and sensitivity to input.”

Line 114
The formulation "rendering our findings applicable to any neocortical region" in line 114 is a very strong statement that remains to be proven. Please reformulate.

We agree with the suggestion. Lines 113-116 now read: “We did not intend to simulate a specific cortical region; instead, we integrated characteristics such as sparse connectivity, inhibition-dominated behavior, and asynchronous irregular firing among neurons, which are commonly observed across the neocortex in awake animals.”

Line 126
"These synapses are randomly established with a (10%)
connection probability." -> "These synapses are randomly established with a connection probability of 10%."

Thank you for the suggestion. This has been implemented and lines 128-129 now read “These synapses are randomly established with a connection probability of 10%.”

**Reviewer #3**

The authors have addressed all my concerns. Thank you very much.

Thank you for your detailed feedback and the time invested in reviewing our manuscript. We are grateful for your insightful comments, which have significantly contributed to enhancing the quality of our work.
